# Development and Optimisation of a Stability-Indicating Analytical Method for Itraconazole in Drug Product by High-Performance Liquid Chromatography Using a QbD Approach

**DOI:** 10.3390/ph19071066

**Published:** 2026-07-10

**Authors:** Alex Fraschi-Nieto, Xavier Mula-Roldán, Lluís Gavaldà-Cánovas, Débora Mercadé-Frutos, Encarna García-Montoya, Marc Suñé-Pou, Pilar Pérez-Lozano

**Affiliations:** 1 Department of Pharmacy and Pharmaceutical Technology and Physical Chemistry, Faculty of Pharmacy, University of Barcelona, Av. Joan XXIII, 27-31, 08028 Barcelona, Spain; alexfraschi@ub.edu (A.F.-N.); xavimula@ub.edu (X.M.-R.); ll.gavalda.c@ub.edu (L.G.-C.); debora.mercade@ub.edu (D.M.-F.); encarnagarcia@ub.edu (E.G.-M.); perezlo@ub.edu (P.P.-L.); 2 Pharmacotherapy, Pharmacogenetics and Pharmaceutical Technology Research Group, Bellvitge Biomedical Research Institute (IDIBELL), Av. Gran Via de l’Hospitalet, 199-203, 08090 L’Hospitalet de Llobregat, Spain

**Keywords:** high-performance liquid chromatography, itraconazole, design of experiments, stability-indicating method

## Abstract

**Background/Objectives:** In this study, an analytical method by high-performance liquid chromatography (HPLC) that allows the determination of the content of the active ingredient itraconazole (ITZ), as well as the quantification of its degradation products and known impurities, is developed. Some of the existing methods, including the compendial ones, present long run times or different methods for the quantification of ITZ and the quantification of its impurities. **Methods:** In this article, an initial screening approach, starting on the described compendial methods, was conducted in order to establish the initial working conditions of the chromatographic method parameters. Once these parametric conditions were established, an optimisation was conducted utilising a statistical approach and a Design of Experiments (DoE), with the objective of attaining an optimised method that allows the quantification of ITZ and its related substances in one single injection. The data obtained during the DoE were processed using the Minitab^®^ statistical programme. Once an optimised method was obtained, a short pre-validation assessment studying the selectivity, linearity and system suitability, among others, was carried out, confirming the suitability of the method for the intended use. **Results:** The resulting method employs an isocratic mobile phase of acetonitrile (ACN) and buffer (ammonium phosphate 10 mM at pH 4.0), utilising a stationary phase of a Poroshell EC-C18 150 × 3.0 mm, 2.7 µm column. The retention time of itraconazole is 8.5–9.0 min, with a total chromatographic run time of 15 min. **Conclusions:** The developed method exhibits a shorter run time and greater environmental sustainability compared to compendial methods, underscoring its improved analytical performance.

## 1. Introduction

Itraconazole is an antifungal drug that belongs to the triazole class of compounds [1]. As with most active pharmaceutical ingredients (API), itraconazole contains several impurities, related to its synthesis process (synthesis impurities, related substances and degradation products). Furthermore, it can undergo degradation under certain conditions, which results in the formation of degradation products. These degradation products can form during manufacturing, transportation and storage of the product, resulting in an impact in the quality, safety and efficacy of the pharmaceutical product. Thus, development of an analytical method that permits the correct quantification of these degradation products in the final product is essential to ensure the final quality, safety and efficacy during its release and throughout its life cycle [2,3].

The official methods for quantifying itraconazole and its impurities are described in the European Pharmacopoeia (EP) [4], which refers to the active ingredient only. The quantification of impurities is achieved through the utilisation of an HPLC system; however, the quantification of itraconazole is accomplished via potentiometry. In the United States Pharmacopeia (USP), which refers to the active ingredient [5] and the final pharmaceutical product, which is available in capsules [6] for human use, the quantification of itraconazole and its impurities by HPLC are described.

The aforementioned methods are characterised by the utilisation of a comparable methodology for the HPLC system, with the employment of a gradient elution system being a common element across them. Gradient HPLC methods are advantageous in that they allow the separation and resolution of most of the peaks of the various components of the analysed sample. This is sometimes necessary, depending on the polarities or behaviours of these products [7,8,9]. However, it should be noted that gradient methods require longer conditioning times before analysis can begin and can generate higher baseline noise [10,11]. Despite the fact that isocratic methods are more challenging to utilise for peak separation, these methods generally require less conditioning time, are more readily transferable, and generally exhibit a superior baseline with reduced noise and interference when the composition is analogous to that of the matrix of the sample to be analysed.

Furthermore, this study aims to reduce the volume of organic solvents utilised in the aforementioned methods and minimise the analysis’s running time, thereby developing a methodology with a reduced environmental impact [12,13,14,15]. This evaluation on the impact will be evaluated by AGREE software (version 0.5), and the obtained score compared to the compendial methods [16].

It is recommended that new analytical methods be developed in accordance with the ICH Q14 [17] and should be founded on a Quality by Design (QbD) framework. The implementation of QbD in the development of an analytical method enhances the method’s robustness by facilitating understanding of the process, risk management and continuous improvement. QbD facilitates the accurate identification of method variables and their impact on performance and quality, thereby ensuring the development of a more reliable and optimised method. This approach involves the utilisation of statistical studies, including Design of Experiments (DoE), for the investigation of various variables and their interrelationships. This methodological approach facilitates a more profound comprehension of the underlying methodology [18,19,20,21,22,23,24,25].

This approximation is not new, and other authors had addressed this optimisation of the method for the determination of itraconazole, in some cases maintaining the gradient and in others applying an isocratic approach. These articles describe stability-indicating methods; however, not all of them identify or quantify the known ITZ impurities listed in the EP [26,27,28,29].

This article employed two approaches for determining and quantifying itraconazole and its impurities. The initial approach involved the screening of different chromatographic parameters, while the second approach consisted of a statistical study of the most impactful parameters. Once these parameters were established, a pre-validation study was conducted to ensure the feasibility of the method for its intended use.

## 2. Results and Discussion

### 2.1. Analytical Target Profile and Critical Method Attributes Study

Initially, the Analytical Target Profile (ATP) was defined to establish the parameters that the developed analytical method was required to achieve. The ATP is a fundamental element in QbD, as it defines the method of purpose and the performance criteria required to ensure reliable analytical results. This process ensures that the final analytical method is fit for the intended purpose [30,31,32,33,34]. In this case, the method must be suitable for the quantification of the active ingredient ITZ and its degradation products [35]. The ATP study, and the rationale for each of the characteristics is shown in Table 1.

With the discussed ATP and its justifications, the Critical Method Attributes (CMAs) were identified. CMAs are the measurable performance characteristics of the analytical method that directly indicate whether the ATP is being met, thus facilitating the measurement and monitoring of the ATP’s achievement. This, in turn, enables the acquisition of the desired characteristics of the chromatographic method [35,36]. The CMA and its rationale are shown in Table 2. 

The following acceptance criteria were applied to the CMA parameters [35,36,37]:

- Symmetry: 0.8–1.8
- Resolution ≥ 1.5
- Retention time: Reproducible between the same method
- Limit of quantification (LoQ): The peak should have a S/N greater than 10, although could be demonstrated experimentally
- Theoretical plates (N) ≥ 2000
- Capacity factor (k’) ≥ 1.0

Following an examination of these parameters, an initial approach to the screening of the various chromatographic conditions was conducted.

### 2.2. Screening Process

The chromatographic methods on which the initial development stages are based are those described in the EP (for the active ingredient), as well as the method described in the USP, also for the active ingredient.

Employing this information, the method described in the EP was conducted at a concentration of 300 µg/mL, as outlined in the USP, creating the method ITRACO01 (all developed methods during the study were named ITRACOXX, where XX is a numerical number in ascending order from the date of creation).

The parameters studied in each of the following discussed methods, ranging from ITRACO01 to ITRACO15, are listed in Tables S1–S7.

In ITRACO01, the retention time of the ITZ bulk product (supplied by SMS Pharmaceutical Ltd., Hyderabad, India) was compared with that of the ITZ CRS batch 3.0, in order to determine whether the bulk product corresponds to ITZ, which is reported to have a retention time of 14 min when using the EP method. The column used in this study was a Zorbax C18 100 4.6 mm, 3.5 µm.

Once the ITZ of the bulk product was identified, the wavelength spectrum of the product was studied with the purpose of determining the most suitable wavelength for the analysis of ITZ. There were two maximum absorption wavelengths: one corresponding to 225 nm and another to 260 nm. The latter wavelength is more pronounced and selective as is further from the initial wavelengths of 190 nm to 200 nm, where most of the solvents used in the sample preparation and HPLC system eluents absorb. The wavelength of 260 nm is more selective because aromatic compounds, which ITZ is formed, absorb at a higher intensity [38,39]. Consequently, the 260 nm wavelength was established as the as the principal wavelength.

In addition to the wavelength of 260 nm, it was decided to change the buffer used from tetrabutylammonium hydrogen sulphate to ammonium dihydrogen phosphate because the former was observed to precipitate during the analysis, possibly reducing the column’s lifespan. The initial salt concentration was set at 10 mM, with the pH adjusted to 2.0. The other chromatographic conditions remained unchanged from those described in ITRACO01. These changes, applied in ITRACO02, led to an increase in the retention time of ITZ, appearing between 15 and 21 min, indicating that the use of ammonium dihydrogen phosphate could lead to the improvement of the resolution between peaks. No significant differences between both wavelengths were observed regarding its areas and heights, establishing the 260 nm as the preferred wavelength.

Nevertheless, the ITZ peak was observed to appear at the midpoint of the phase change of the gradient, which could lead to an increased variability of the method. Consequently, the gradient conditions were modified, creating the method ITRACO03. With the new developed method, the ITZ peak appeared during the stabilised phase of the gradient at a proportion of 50:50 BS:ACN.

It was decided to eliminate the gradient of the method and study the use of an isocratic mobile phase with the proportions of 50:50 (ITRACO04), 45:55 (ITRACO04A) and 40:60 (ITRACO04B) ACN:BS. All obtained ITZ peaks presented correct symmetries and forms, but it was observed that for each reduction in the proportion of ACN (5% each), the retention time varied in 2–3 min, appearing at 2.0, 4.8 and 7.2 min, respectively. This observation serves to highlight the impact of ACN in the present chromatographic system and ITZ’s elution.

As an isocratic method was being used, a decision was taken that the diluent to be employed for the preparation of the reference and the sample solutions should be the same as the mobile phase. This was done in order to improve the symmetry, the retention time and the selectivity of the chromatographic system. The pH of the buffer solution (BS) was adjusted to 2.5, which is closer to the pKa of the active ingredient (3.7) [40]. These changes resulted in the creation of ITRACO05 method.

Under these chromatographic conditions, samples at the working concentrations of 1 and 100 µg/mL, for related substances and ITZ quantification respectively, were prepared and injected at different volumes, to establish the most suitable for the correct quantification of each concentration.

The results obtained are presented in Table 3.

In both studied concentrations, the peaks exhibited adequate symmetry and height at 20 µL, thus establishing this volume as the optimal injection parameter.

**Figure 1:**
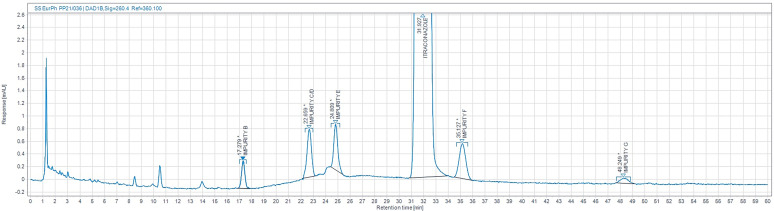
Chromatogram of itraconazole for System Suitability CRS.

**Figure 2:**
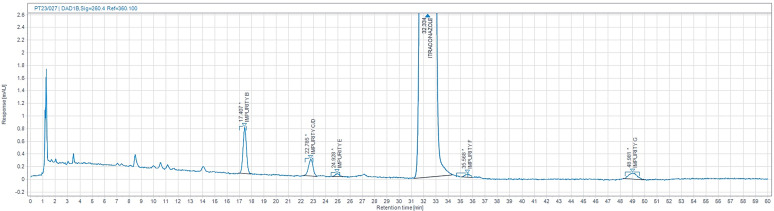
Chromatogram of itraconazole from SMS Pharmaceuticals Limited.

With this same method, two samples were prepared: one with ITZ for System Suitability CRS (see Figure 1), and another with ITZ raw material from SMS Pharmaceuticals Limited (see Figure 2).

The impurities present in the System Suitability sample from EP correspond to those observed in the ITZ raw material sample.

In order to enhance the selectivity of the process, a new column was utilised that featured a more deactivated type of C18 being a Mediterranea SEA C18 150 × 4.6 mm, 3.0 µm [41,42]. This alteration to the methodology resulted in the creation of ITRACO06 which made the ITZ peak appear at 40 min with a symmetry of 1.14.

Minor alterations were implemented on ITRACO06 in order to adjust the retention time and symmetry with the new column:

- ITRACO06A: 55:45 (ACN:BS)
- ITRACO06B: Flow rate: 1.5 mL/min
- ITRACO06C: 52:48 (ACN:BS)
- ITRACO06D: Flow rate: 1.2 mL/min

**Figure 3:**
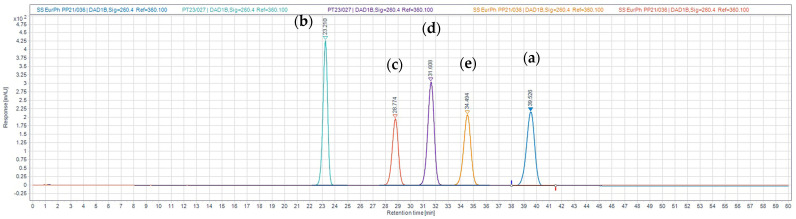
Chromatograms obtained in the study of ITRACO06 from A to D: (a) being 06; (b) being 06A; (c) being 06B; (d) being 06C; and (e) being 06D.

The obtained chromatograms are presented in Figure 3.

ITRACO06C exhibited comparable retention times to ITRACO05; however, it demonstrated superior resolution and baseline performance. The resolution between impurities was lower in ITRACO06A and as a result, it was discarded. In the case of ITRACO06B, a resolution analogous to ITRACO06 was obtained, but with a lower retention time. ITRACO06D demonstrated a higher retention time, consequently being eliminated from further consideration.

In consequence of the results obtained, a new column with a C18 similar to the Mediterranean SEA but shorter in length was tested. The column employed for this analysis was an ODS Hypersil 100 × 4.6 mm, 3.0 µm (ITRACO07), maintaining the other conditions as in ITRACO06B, and modifying the flow rate set at 1.2 mL/min (ITRACO08). There was no enhancement in resolution, which prompted the discarding of the ODS Hypersil 100 × 4.6mm, 3.0 µm column and the study of columns Luna Phenyl-Hexyl 150 × 4.6 mm, 5 µm (ITRACO09) and Dionex Acclaim C18 33 × 3.0 mm, 3 µm (ITRACO10). Both columns resulted in lower resolutions between the peaks, with the Phenyl-Hexyl having a more unstable and noisier baseline; for this, both columns were discarded.

An Acquity CSH C18 100 × 3.0 mm, 1.7 µm, with a reduced particle size was tested (ITRACO11). The symmetry of the ITZ peak was found to be 0.99, and the resolution between ITZ and impurity F peak was improved. Despite these results, the pressure within the chromatographic system was found to be excessively high (320 bar), thereby reducing the operational lifespan of the column. Consequently, a larger particle size and a greater column diameter were employed, utilising the Poroshell EC-C18 100 × 4.6 mm, 2.7 µm column. This change in the column was accompanied by the study of three different flow rates (ITRACO12, 13 and 14), being 1.0, 1.2 and 1.5 mL/min. The chromatograms obtained demonstrated low levels of repeatability and symmetry, thus leading to the discarding of the column.

An additional column, characterised by the same stationary phase but distinct measurements, namely the Poroshell EC-C18 150 × 3.0 mm, 2.7 µm, was subjected to testing. This process involved adjusting the flow and injection volume, thereby establishing the ITRACO15 method.

**Figure 4:**
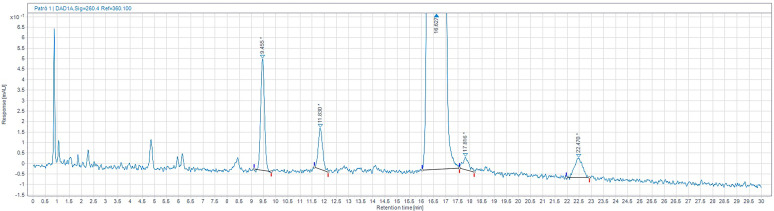
Chromatogram obtained with ITRACO15.

The chromatographic method was found to achieve adequate resolution between the impurities and the ITZ peak, although presenting a certain baseline noise, as seen in Figure 4. These results established this method as suitable for the ITZ assay, as well as for the determination and quantification of its impurities.

A Design of Experiment (DoE) was subsequently executed utilising the statistical software Minitab^®^, version 21.4.2, for the purpose of investigating the optimal chromatographic parameters for the analysis of ITZ and its degradation products.

### 2.3. Optimisation of the Chromatographic Method Using a Statistical Approach

Applying the principles of ICH Q14 enhances the understanding of both the analytical process and product behaviour under varying conditions. This integrated framework, increasingly reflected in recent studies across diverse analytical methods, facilitates the translation of theoretical developments into practical applications [43,44,45,46,47].

In this context, a QbD strategy was implemented for the optimisation of the process, beginning with an initial risk assessment to define the key parameters for evaluation. Based on the previous ATP (Table 1), the CMAs of the chromatographic method were identified as follows:

- Resolution between itraconazole peak and its impurities;
- Itraconazole peak symmetry.

Taking these CMAs into consideration, the critical parameters of the method were studied using a heat map (Table 4), with the parameters marked in red having a higher impact, those marked in yellow having a medium impact, and those marked in green having a lower impact.

The resolution of the peaks is greatly impacted by the concentration of the buffer due to its ionic strength. The greater the ionic concentration in the mobile phase, the greater the interaction between the analyte and the stationary phase, altering its resolution. In terms of symmetry, the impact is moderate due to the aforementioned interactions, but not to the same extent as with other parameters [48,49].

The pH of the buffer solutions significantly impacts resolution and symmetry because it affects the ionisation state of the analyte, thereby altering its retention time and resolution. Additionally, it can lead to partial ionisation, resulting in peak tailing and poor symmetry [50,51].

The impact of the flow rate is moderate because, while the retention times of the peaks will change, the resolution will remain similar. Regarding symmetry, the flow rate can affect peak shape, but this is less significant than other parameters.

The proportion of the mobile phase eluent significantly impacts both attributes because it modifies the elution of the analytes. This is particularly important in this case, as the analyte is highly sensitive to changes in the proportion of the eluent [52,53].

The impact of the stationary phase on both attributes is also critical, as changes to the phase’s chemistry or dimensions will alter the interaction between the analyte and the column [54].

The injection volume has a moderate effect on symmetry, but not resolution, because a saturated peak resulting from a large sample injection produces an undesirable peak shape. Injection volumes above 5 µL are not recommended for the column properties [55].

Inadequate sample preparation can result in an incompatible sample matrix, leading to interactions with the stationary phase or changes in pH that can alter the chemical behaviour of the analytes in the system. For its complexity, and because of the aim of having the same diluents as the mobile phase used in the chromatographic system, this parameter was not studied.

Higher temperatures result in a lower viscosity of the mobile phase. This leads to faster elution through the stationary phase and a lower resolution between peaks. In the case of symmetry, however, the faster elution will not significantly affect the peak shape. This parameter was not investigated in this study because the current temperature of 50 °C keeps the system pressure within the acceptable range of 150–180 bar, thereby extending the column’s useful lifespan. Reducing the temperature would increase the pressure, which could damage the column. Increasing the temperature above 60 °C would also damage the column.

The parameter intervals that were studied in the DoE are described in Table 5.

The intervals studied are based on the final method (ITRACO15) established in the initial screening, with the medium level being the conditions of the aforementioned method, except for the pH. The flow rate variation is studied between ± 0.2 mL/min because the columns tested had a low volume and therefore higher flow rates will result in high working pressures, which will damage the column. The variation of the buffer concentration is ±5 mM from the medium condition because higher concentrations of salt, considering the high proportion of ACN in the mobile phase, could result in a precipitation of the salt, also damaging the column. The proportion of eluents in the mobile phase only has a variation of a 5% of each component because during the initial screening it was observed the great impact of the ACN proportion in the ITZ retention. Finally, the pH was decided to be studied at the interval between 3 and 5 because the pKa of the ITZ is 3.7 [40], being in this case the medium condition.

These factors were optimised through a full factorial statistical study of five factors at two levels, including central points and two replicates (the experiments conducted are listed in Table S9). The obtained experimental results (the results for the experiments conducted in DoE are expressed in Table S10) were analysed using Minitab^®^.

In all cases, the obtained symmetries were close to 1.0, so this parameter was deemed not significant. Instead, the resolution and retention time of the peaks varied greatly depending on the conditions.

The statistical evaluation of the results by ANOVA (Table S8) showed that all studied parameters were significant for both CMA (*p* < 0.05). All two-factor interactions also showed significance in both CMA items, except for mobile phase and buffer concentration for the retention time. Observing the F-values, it was possible to determine that for the retention time the flow rate and mobile phase were more influent, while in the resolution the most influent parameter was the mobile phase, followed by the column. In both cases the buffer concentration presented the lowest impact. All two-factor interactions were significant except for the mobile phase and the buffer solution concentration for the retention time.

For the model applied to the retention time, a lack-of-fit was observed, with a *p*-value of 0.007, but the low error (0.002) and the above 0.99 in R^2^, R^2^ adjusted and R^2^ predicted determined that the results obtained in the study for this parameter were statistically significant. For the model applied to the resolution, a lack-of-fit value of 0.336 was obtained, with an error of 0.001 and R^2^, R^2^ adjusted and R^2^ predicted above 0.99 being a good fit.

A study using contour graphics was conducted to determine the operating range at which the obtained parameters would be as optimal as possible given the observed experimental conditions.

Studying the effect of the parameters on the resolution, it was observed that a flow rate greater than 0.5 mL/min achieves a resolution between 2.5 and 3.0 for most mobile phase proportions. For a 50:50 ACN:BS and the lowest flow rate of 0.3 mL/min, the maximum resolution was between 3.0 and 3.5, but this also implies a high retention time of 25–30 min and a longer chromatogram.

**Figure 5:**
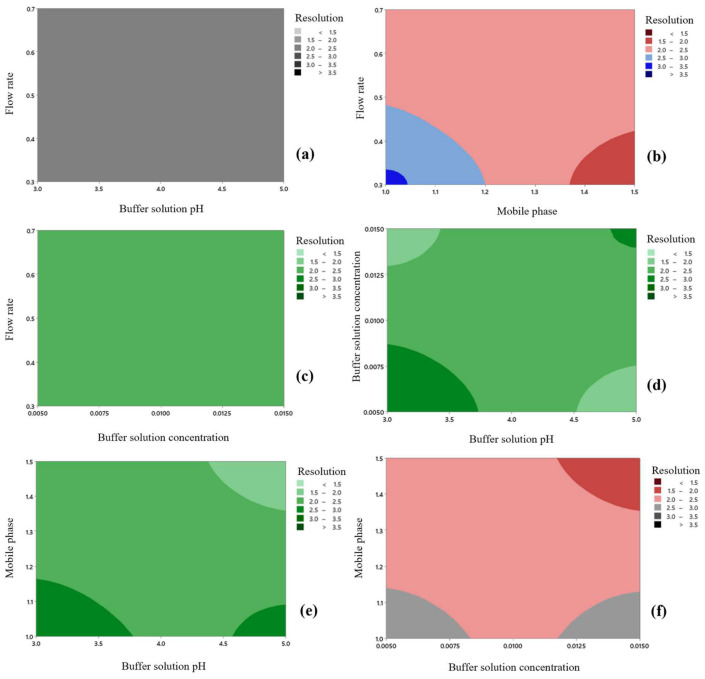
Contour graphics studying the resolution between ITZ and impurity F. (**a**) Flow rate vs. buffer solution pH; (**b**) flow rate vs. mobile phase proportion; (**c**) flow rate vs. buffer solution concentration; (**d**) buffer solution concentration vs. buffer solution pH; (**e**) mobile phase proportion vs. buffer solution pH; (**f**) mobile phase proportion vs. buffer solution concentration. The mobile phase proportion scale described in the contour graphics is the relation between ACN and BS (ACN/BS), therefore 1.2 equals to 55:45 ACN:BS.

As illustrated in Figure 5, buffer concentration does not greatly affect the retention of the product, with optimum results being achieved near to the central value of 10 mM. A similar trend was observed for the buffer solution pH: the closer the pH value is to the central point of 4.0, the better the resolution. However, a tendency for a lower pH to result in a better resolution than a higher pH was also observed.

**Figure 6:**
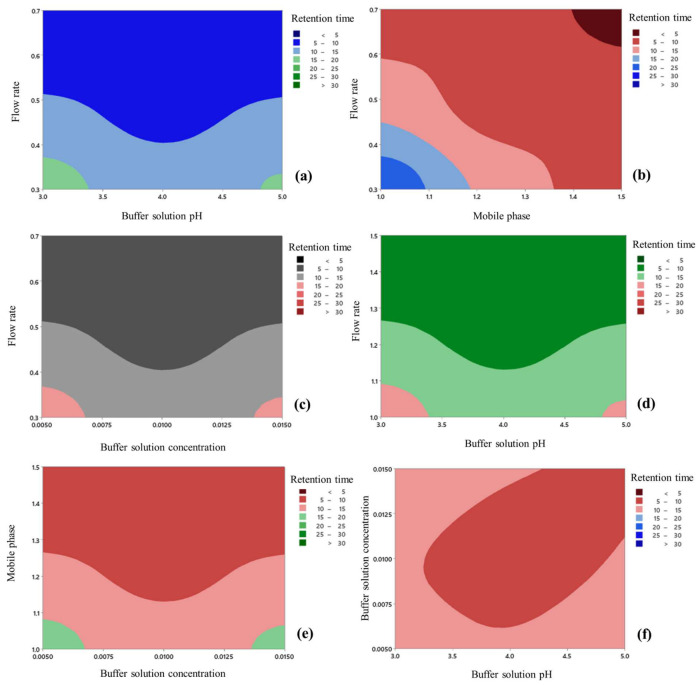
Contour graphics studying the retention time of ITZ. (**a**) Flow rate vs. buffer solution pH; (**b**) flow rate vs. mobile phase proportion; (**c**) flow rate vs. buffer solution concentration; (**d)** mobile phase proportion vs. buffer solution pH; (**e**) mobile phase proportion vs. buffer solution concentration; (**f**) buffer solution concentration vs. buffer solution pH. The mobile phase proportion scale described in the contour graphics is the relation between ACN and BS (ACN/BS), therefore 1.2 equals to 55:45 ACN:BS.

As illustrated in Figure 6, the higher the proportion of ACN in the mobile phase, the lower the retention time of ITZ; however, this reduces its resolution. The same occurs with the flow rate: the higher the flow rate, the lower the retention time, but the column pressure increases. Buffer concentration does not greatly affect retention of the product, with optimum results being achieved nearer to the central value of 10 mM. A similar trend was observed for the pH of the buffer solution: the closer the pH value is to the central point of 4.0, the better the resolution.

Based on these results, a second Design of Experiments (DoE2) was carried out to optimise the chromatographic method. Considering the outcomes of the initial DoE, the column was fixed at Poroshell EC-C18 150 × 3.0 mm, 2.7 µm, as it provided the best overall performance. Likewise, the buffer concentration was set at 10 mM, since no significant differences were observed among the concentrations previously evaluated. In this second DoE, the resolution was selected as the sole response variable for optimisation. The studied parameters were the flow rate, in a more reduced interval from 0.4 to 0.6 mL/min; the mobile phase, which in the previous DoE was observed to have a great impact on both the retention time and the resolution; and the buffer pH, also with a more reduced interval spanning from pH 3.5 to 4.5.

As in the first DoE, DoE2 was a full factorial design with three variables and two levels, including a central point (the experiments conducted are listed in Table S11). Once the experiments were finished (the results for the experiments conducted in DoE2 are shown in Table S12), a statistical analysis was conducted using Minitab^®^.

It was concluded that the proportion of the mobile phase was the only parameter affecting the resolution. However, the statistical model showed that the results were not linear, indicating that an additional curvature approximation was required to adjust the model correctly (additional experiments for the curvature approximation are listed in Table S13 and the obtained results are shown in Table S14).

**Figure 7:**
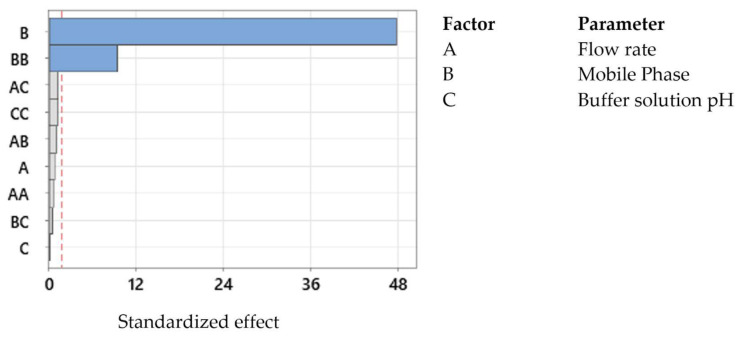
Pareto’s diagram for DoE2. The red dashed vertical line indicates the statistical significance threshold. Bars exceeding this threshold are coloured blue, highlighting statistically significant effects, whereas grey bars indicate non-significant effects.

The new quadratic model with an R^2^ of 0.9883 shows that the proportion of the mobile phase is the only factor affecting the resolution, as represented in the Pareto’s diagram (Figure 7).

**Figure 8:**
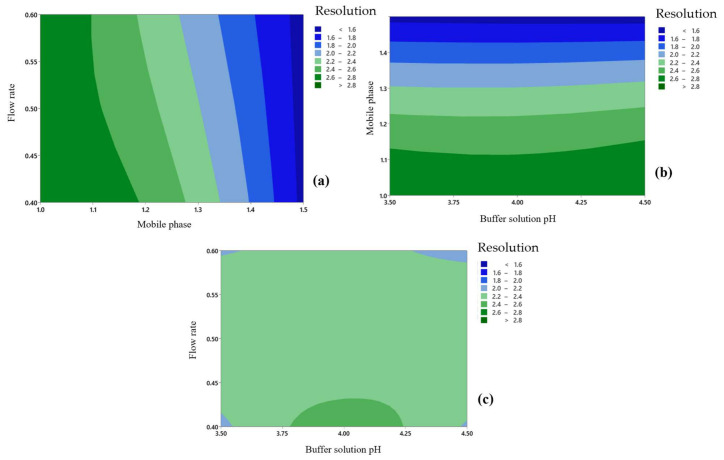
Contour graphics from the second DoE (DoE2) corresponding to: (**a**) flow rate vs. mobile phase proportion; (**b**) buffer solution pH vs. mobile phase proportion; (**c**) buffer solution pH vs. flow rate. The mobile phase proportion scale described in the contour graphics is the relation between ACN and BS (ACN/BS), therefore 1.2 equals to 55:45 ACN:BS.

Contour graphics were studied to confirm that neither the pH nor the flow rate had an influence on the resolution (see Figure 8).

No great impact was observed by the flow rate and the buffer solution pH alone, confirming that these two parameters do not present a significant effect on the studied parameters. Studying the contour graphics corresponding to the mobile phase, it was observed that the best proportion of the mobile phase was 55:45 ACN:BS, achieving a resolution of 2.4–2.6. This produced a faster chromatogram with an acceptable resolution.

The flow rate was set to 0.6 mL/min to produce a faster chromatogram while maintaining pressure control. The pH range was set to 4.0 as this is closer to the pKa of ITZ (3.7) and less acidic than 3.5, which would damage the column and cause silanol degradation products [56,57].

Based on these results, the method described in Table 6 was established.

### 2.4. Selectivity Study of the Optimised Analytical Method

**Figure 9:**
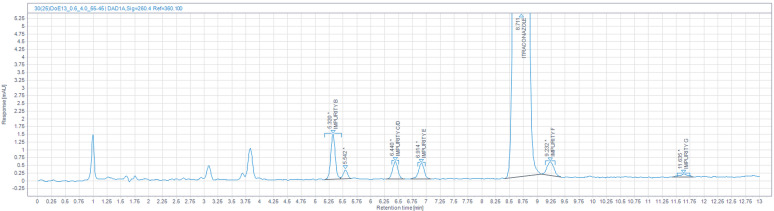
Chromatogram obtained with the optimised chromatographic method using a reference solution containing ITZ and its known impurities as described in the EP.

This new optimised method was tested by preparing a reference solution containing the ITZ and its known impurities described in the EP at a ITZ concentration of 100 µg/mL. The following chromatogram (Figure 9) was obtained:

The main ITZ peak appears at a retention time of 8.7 min, with a symmetry value of 1.04. All known impurities have a resolution value greater than 1.5; impurity F has a resolution value of 2.2. All capacity factors are above 1.5, and the number of theoretical plates is above 2000. This indicates that the method provides good retention and high separation efficiency for the studied peaks. These studied known impurities are identified by comparing its retention time to the ones observed in the sample of System Suitability from the European Pharmacopoeia; further studies to corroborate the peak identification should be carried out.

In Table 7 the results corresponding to resolution (Rs), capacity factor (k’) and number of theoretical plates (N) are outlined.

A stress study was conducted to determine the method’s capacity to quantify and separate the various potential degradation products. This study was conducted in accordance with the recommendations of ICH Q1A(R2) [58], under the conditions described in Section 3.5. The chromatograms obtained for acidic hydrolysis after 72 h for the active ingredient (Figure S1) as well as for a solid form for oral administration containing ITZ (Figure S2) showed good resolution between the degradation peaks and ITZ peak.

The stress conditions for alkaline hydrolysis, oxidative medium and temperature were also studied. This study was conducted using both API and the finished product (FP) developed in the SDM. The results obtained are shown in Table 8, along with the mass balance (MB) calculations.

Good resolution was achieved between the degradation peaks and ITZ peak.

The stress conditions for alkaline hydrolysis, oxidative medium and temperature were also studied. This study was conducted using both API and the FP in a solid oral dosage form developed in the SDM.

All results correspond to maintaining the stress conditions for 72 h, except for the analysis of oxidative conditions, which was conducted after 24 h. This difference in the study time was due to the higher degradation observed in the oxidative media, in which at 24 h more than a 50% was degraded in the finished product.

Observing the MB, calculated by the summatory of the degraded ITZ peak and the observed impurities, a balance between 95–105% is obtained in all conditions except for the FP in the oxidative media, in which more than a 90% of ITZ was degraded. Despite this extreme degradation, a MB of 77.08% was obtained. This degradation difference between the API and the FP is probably because the active ingredient in the FP has suffered a change in its configuration due to the manufacturing process.

Acceptable resolution was obtained between the observed degradation products, the known impurities, and ITZ. Of the known impurities, impurities B and E were observed to increase during stress study under alkaline conditions for impurity B and temperature, acidic hydrolysis and oxidative conditions for impurity E. It was therefore concluded that, in addition to being synthesis impurities, they are also degradation products. As a result, impurities B and E will be monitored in further stability studies, along with the listed unidentified degradation products listed in Table 8, once a finished product of ITZ is manufactured.

An initial estimation of the LoQ was conducted using the obtained S/N from a sample prepared at the established working concentration for degradation products of 1.0%. The obtained S/N value was of 70.25 with a sample concentration of 0.992 µg/mL; the following equations were applied to obtain the theoretical LoQ:

(1) $$LoQ=\frac{10}{S/N}\times Sample\;concentration;$$

With this equation, a theoretical LoQ of 0.14 µg/mL was obtained. With the same data, the limit of detection (LoD), was also calculated according to the following equation:

(2) $$LoD=\frac{3}{S/N}\times Sample\;concentration;$$

obtaining a theoretical LoD of 0.04 µg/mL.

The LoD and LoQ were experimentally demonstrated in the feasibility of the method study in 2.5.

### 2.5. Method Feasibility Study

To determine the feasibility of the method, for its intended purpose as a stability-indicating method, an initial pre-validation study, with some of the described parameters in the ICH guideline Q2(R2) [35] was conducted. The studied parameters were the system suitability of the reference solution, the stability of the reference solution at quantification and related substances concentration, and the finished product, the linearity, the accuracy with a placebo solution spiked with ITZ, the repeatability of the reference solution and finished product solution, the intermediate precision for the standard solution at the quantification concentration and related substances concentration and for the finished product solution and, only in the case of the related substances concentration, the study of the LoD and LoQ. According to ICH Q14 [17] it is not necessary to repeat experiments for the method robustness if during the method development a robustness data from a DoE is obtained, which is the case of the present method. During development, the working space of different critical parameters, like the buffer pH, concentration, and flow rate, were studied and defined, not requiring further study in this step of the feasibility of the method.

The obtained results for the quantification of ITZ are shown in Table 9, and for its impurities and degradation products in Table 10.

It was concluded that the proposed method, for the quantification of ITZ, is linear, accurate and repeatable from the concentrations between 70 and 130 µg/mL, establishing this range as the working range. For the quantification of ITZ impurities and degradation products, it was concluded that the method is linear, accurate and repeatable from 0.15 to the 1.30 µg/mL concentration, establishing this concentration range as the working one for the method. Both theoretical LoD and LoQ concentrations were corroborated experimentally during the feasibility study of the developed method. The tested solutions are repeatable and precise in the studied variation and are stable for 48 h from its preparation.

Once this method feasibility was finished, it was applied to a finished product in tablets for oral administration, with the sample preparation as described in Section 3.4. A recovery of 100.62%, with a 0.59% of RSD, with three tested samples was obtained. Also, known impurities B, C, D and G were observed but at concentrations below the LoQ; no other degradation products were observed. The method is therefore suitable for the quantification of ITZ and its related substances in the intended finished product in tablets for oral administration and to possess stability-indicating capability.

No interactions were observed between the reference and finished product solutions with the placebo solution (see Figure S3), where no peak seems to interfere in the correct study of ITZ and its related substances.

### 2.6. Method Comparison with Current Technology

The developed and validated method in the present article was compared with similar articles in which a stability-indicating method are developed, as summarized in Table 11.

As seen in the described articles, only in the article by Agrawal et al. were all the impurities described in the EP monograph studied, and more. This article uses a gradient eluent configuration and is applied into an ultra-high-performance liquid chromatography (UPLC) system. For Arghode et al. which, as Agrawal et al. and the developed method in this article, which applies the QbD, the authors developed an HPLC method with an isocratic elution, but in this method triethylamine (TEA) is used, which is toxic compared with the solvents and reagents used in the present developed method. Also, the impurities described in the studies of forced degradation did not correspond to the ones described in the EP, not being equivalent to the compendial method.

The developed method presents LoD and LoQ similar to Kasagić et al. and Agrawal et al. but the former only characterises two of the described impurities (B and F) and the latter presents a higher run time of 25 min. Despite Reddy et al. having developed a fast chromatogram of only 7 min, and studying the LoD and LoQ, no study of the detected impurities was done.

A similar comparison, summarized in Table 12, was conducted with the developed method and the compendial methods from EP [4] and USP [5,6]. This table also indicates the AGREE score, applied to the developed method, and the compendial ones using the AGREE software [16].

As discussed previously, the described HPLC method in EP is only intended for the quantification of impurities, and not for the assay of ITZ, which is carried by potentiometric titration. The EP method and the USP method for related substances are almost the same, only varying the sample preparation process. Both these methods needed a high ITZ concentration of 10,000 µg/mL and use a high concentration of tetrabutylammonium in the buffer solution (80 mM). Also, both methods had a run time of 27 min. The compendial method for the finished product in capsules has a run time of 50 min, also with a gradient, and uses tetrahydrofuran (THF) in the mobile phase and diluent, which is more toxic than the other used solvents.

**Figure 10:**
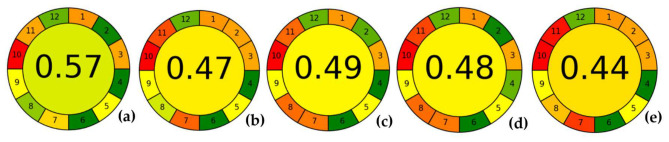
AGREE score diagrams. (**a**) Developed method in the present study; (**b**) European Pharmacopoeia and United States Pharmacopeia methods for itraconazole known impurities; (**c**) United States Pharmacopeia method for the assay of itraconazole; (**d**) United States Pharmacopeia method for the assay of itraconazole in capsules; (**e**) United States Pharmacopeia method for itraconazole known impurities in capsules. Each circular diagram consists of 12 sectors corresponding to the technical parameters considered in the greenness assessment (described in Pena-Pereira et al. [16]). The colour of each sector reflects the environmental performance of the corresponding parameter, with green indicating a more environmentally friendly approach and red indicating a less sustainable one. The numerical value at the centre of each diagram represents the overall greenness score obtained from the combined evaluation of all 12 parameters.

The developed method presents a lower run time of 15 min with a single-step sample preparation and run, that can quantify ITZ and its related substances. Also, the flow rate is lower than in the compendial methods. These changes indicate that the developed method presents a higher AGREE score (higher environmental sustainability) than the compendial methods, as illustrated in Figure 10. The method by Arghode et al., which also studies the Ambiental impact through the AGREE score, presents a score of 0.72, indicating a higher environmental sustainability than the present method, but without the identification of the compendial described known impurities.

## 3. Materials and Methods

### 3.1. Materials

The reference materials were: Itraconazole CRS batch 3.0 and Itraconazole for System Suitability CRS batch 4.0 from the EP; and Itraconazole batch ITZ/0120022 from SMS Pharmaceuticals Limited (Hyderabad, India).

All reagents utilised in the present study were of HPLC quality and procured from PanReac Química S.L.U. (Castellar del Vallés, Spain). The reagents in question were as follows: tetrabutylammonium hydrogen sulphate; acetonitrile; ammonium dihydrogen phosphate; hydrochloric acid 37%; and methanol.

### 3.2. LC-DAD System

The Agilent 1200 HPLC system, manufactured by Agilent Technologies Inc. (Santa Clara, CA, USA) was employed for the development and optimisation of the method under discussion in this study. The instrument consists of a quaternary pump (G1311A), a degasser (G1322A), an automatic sampler (G1329A), a column oven (G1316A), and a diode array and multiple wavelength detector (G1315D).

In the course of the study, a variety of chromatographic columns (stationary phase) were employed, including: Zorbax C18 100 × 4.6 mm, 3.5 µm; XSELECT HSS T3 C18 250 × 4.6 mm, 3.5 µm; Mediterranea SEA C18 150 × 4.6 mm, 3.0 µm; ODS Hypersil 100 × 4.6 mm, 3.0 µm; Luna Phenyl-Hexyl 150 × 4.6 mm, 5 µm; Dionex Acclaim C18 33 × 3.0 mm, 3 µm; Acquity CSH C18 100 × 3.0 mm, 1.7 µm; Poroshell EC-C18 100 × 4.6 mm, 2.7 µm; Poroshell EC-C18 150 × 3.0 mm, 2.7 µm.

### 3.3. Reference Preparation

All reference solutions that were prepared during the study were made using the method described for each stage of development and optimisation.

### 3.4. Sample Preparation

All samples during the screening part were prepared weighing the required quantity of ITZ in a volumetric flask to obtain the specified concentration of ITZ. The used diluent for the correct dissolution of the API was methanolic hydrochloric acid for the samples prepared from ITRACO01 to ITRACO04B and the mobile phase of the chromatographic method from ITRACO05 to ITRACO15.

The general preparation process was conducted as follows: Weigh the required amount of ITZ into a volumetric flask of a specified volume. Add about 80% of the diluent and sonicate for 10 min. Allow to cool down and bring to the final volume with more diluent. Homogenised the solution obtained with magnetic stirring.

For the finished product in tablets, containing ITZ, shred a minimum of 3 tablets and weigh the equivalent quantity of the finished to obtain 10 mg of ITZ into a 100.0 mL volumetric flask. 80 mL of diluent and sonicate for 10 min. Allow to cool down and bring to the final volume with more diluent. Homogenise the solution obtained with magnetic stirring.

### 3.5. Sample Preparation for Stress Study

In accordance with the recommendations outlined in ICH Q1A(R2) [58], the stress study was conducted employing the following sample preparation methodology detailed in Table 13, with every sample having a concentration of ITZ equivalent to 100 µg/mL. The FP solutions methodology is expressed as an example case where the final formulation has a concentration of 20% of ITZ.

### 3.6. Acceptance Criteria

The results obtained were partially validated and compared with the validation characteristics described in the ICH Q2(R2) [35] guideline and in the general chapters of the EP [36]. The parameters of the method that were the subject of study included linearity, the limit of quantification, selectivity and system suitability.

### 3.7. Statistical Study

The statistical development of the optimisation process was conducted utilising Minitab^®^ edition 21.4.2 software, with which a Design of Experiments (DoE) was established. Following the conclusion of all conducted experiments, the resulting data were subjected to statistical analysis.

## 4. Conclusions

A new high-performance liquid chromatography stability-indicating method for the quantification of ITZ and its impurities and degradation products has been developed. The following chromatographic conditions, as listed in Table 6, were established: a stationary phase Poroshell EC-C18 150 × 3.0 mm, 2.7 µm; mobile phase composed of a mixture of 55% of ACN and 45% of 10 mM ammonium dihydrogen phosphate (NH_4_)(H_2_PO_4_) buffer solution in water adjusted to pH 4.0 at a flow rate of 0.6 mL/min; wavelength of 260 nm; injection volume of 5 µL; a column heater temperature of 50 °C; a run time of 15 min; and a working concentration of 100 µg/mL of ITZ. The diluent used in preparing the sample and reference solutions is qualitatively the same as the mobile phase, but at a proportion of 50:50 ACN and BS.

This method has a working range for ITZ quantification from 70 to 130 µg/mL and from 0.15 to 1.30 µg/mL for its related substances.

The developed stability-indicating method permits the correct quantification of ITZ and its related substances and is suitable to the intended finished product in tablets for oral administration, all in a shorter run time of 15 min and with a greener method, with an AGREE score of 0.57 instead of 0.47 for the compendial method.

The application of the QbD permitted the more insightful study and compression of the method parameters and the behaviour of the ITZ and its impurities in the chromatographic system.

Further investigation of the method in a more efficient liquid chromatographic systems, like an UPLC, could benefit its sustainability, permitting the obtention of lower run time method but maintaining the resolution and characteristics of the present developed method.

## Figures and Tables

**Table 1:** ATP study and rationale [17,35].

Performance Characteristics	Acceptance Criteria	Rationale
Accuracy	97–103% of average recovery for ITZ in assay studies. A 95–105% of average recovery in degradation products is proposed.	The quantification of ITZ is provided in an unbiased manner, with a 3% acceptable margin. For degradation products a bias of 5% allows to determine correctly the 1.0% established as the acceptance criteria for degradation products in the final product. This bias results in a 0.05% variation, not affecting the analysis results.
Precision	Relative standard deviation (RSD) (%): ITZ (assay) ≤ 1.9% Impurities ≤ 5.0%	An RSD of equal to or less than 5.0% for impurities ensures an acceptable variation between the obtained results. In the case of the ITZ assay, a value of less or equal to 1.9% is established.
Specificity	The analytical method should be capable of quantifying ITZ and its impurities with no undue interference.	Interferences with the ITZ or its degradation products will consequently lead to an erroneous quantification of the components.
Reportable Range	For ITZ in assay at least from 70% to 130%. For degradation products at least from limit of quantification (LoQ) to 1.30%.	The reporting threshold for content uniformity is established at 70–130%, including the 80–120% range for assay. The lower limit for impurities is established at the LoQ established during the method development. The upper limit is fixed at 130%, above the recommended 120% of the acceptance criteria.

**Table 2:** CMA study and rationale [35,36].

ATP Parameter	Supporting CMA	Rationale
Accuracy	Theoretical plates (N) Resolution (Rs) Symmetry	High theoretical plates imply a more efficient chromatographic system, allowing a greater peak separation with narrower peaks Ensuring good resolution is essential to prevent the analyte from co-eluting with other peaks, thus ensuring accurate quantification. The symmetry facilitates the integration of the peak in a reliable manner, thereby ensuring that it does not interact with other factors in the studied peak.
Precision	Resolution Symmetry Signal-to-Noise (S/N)	The reduced variability of peak shapes defined by the symmetry permits a more robust peak integration. Resolution is critical to ensure that the analyte does not co-elute with other peaks. S/N assures that the system noise does not interfere with the area of the peak, assuring a correct system precision.
Specificity	Capacity factor (k’) Resolution Retention time Symmetry	Capacity factor ensures the analyte is retained in the system for enough for its adequate separation. As with *Accuracy* and *Precision*, acceptable resolution and symmetry ensure differentiation of the studied peak and other peaks of the chromatographic system. The identification of the peak is possible through the consistent retention time.
Reportable Range	Signal-to-Noise Resolution Symmetry	A correct S/N would permit the quantification of lower peak concentrations, allowing to establish a lower limit of quantification (LoQ). *Resolution* and *Symmetry* as explained in *Accuracy*.

**Table 3:** Injection volume study results for the analytical method ITRACO05.

Injection Volume (µL)	Area (mAU × s)	Symmetry	Height (mAU)
100 µg/mL			
10	2992.04	0.84	204.7
15	4496.88	0.87	292.2
20	5990.34	1.00	344.2
25	7488.76	1.31	364.9
30	8996.37	1.84	368.3
40	*	*	*
50	*	*	*
1 µg/mL			
10	19.48	0.80	1.4
15	29.48	0.82	2.0
20	36.36	0.90	2.3
25	35.43	0.98	2.2
30	31.89	1.00	2.0
40	25.75	0.92	1.8
50	30.61	0.91	2.1

* The obtained peaks exhibited significant fronting, indicating an overload from the sample, and were therefore excluded from the study.

**Table 4:** Risk assessment study for the DoE in the optimisation process ^1^.

Critical Parameters of the Method	Resolution	Symmetry
Buffer concentration	High	Medium
Buffer pH	High	High
Flow rate	Medium	Medium
Mobile phase	High	High
Stationary phase	High	High
Injection volume	Low	Medium
Sample preparation	High	High
Temperature	Medium	Low

^1^ Cell colours denote the level of parameter criticality: low (green), medium (yellow), and high (red).

**Table 5:** Studied parameters and intervals in DoE.

Parameter	Low	Medium		High
Flow rate (mL/min)	0.3	0.5		0.7
Mobile phase (ACN:BS)	50:50	55:45		60:40
Buffer concentration	5 mM	10 mM		15 mM
Buffer solution pH	3	4		5
Stationary phase	Poroshell EC-C18 150 × 3.0 mm, 2.7 µm		Poroshell EC-C18 100 × 4.6 mm, 2.7 µm	

**Table 6:** Chromatographic conditions for the optimised method by DoE.

Parameter	Condition
Stationary phase	Poroshell EC-C18 150 × 3.0 mm, 2.7 µm
Mobil phase	ACN:BS (55:45)
Buffer solution	10 mM ammonium dihydrogen phosphate at pH 4.0
Flow rate	0.60 mL/min
Wavelength	260 nm
Injection volume	5 µL
Temperature	50 °C
Run time	15 min
Diluent	ACN:BS (50:50)
Concentration	100 µg/mL

**Table 7:** Results obtained with the optimised method for resolution, capacity factor and number theoretical plates of the studied peaks.

Peak	Rs	k’	N
Impurity B	-	3.53	21284.71
Impurity C/D	5.62	4.49	21843.00
Impurity E	2.64	4.89	22118.53
Itraconazole	8.77	6.42	23885.51
Impurity F	2.23	6.86	23465.00
Impurity G	9.29	8.91	27978.19

**Table 8:** Stress study results for the API and FP at acidic and alkaline hydrolysis, temperature and oxidative medium.

Component (%)	Relative Retention Time	Acidic Hydrolysis		Alkaline Hydrolysis		Temperature		Oxidative Medium	
		API	FP	API	FP	API	FP	API	FP
ITZ	1.00	98.51	94.89	103.12	92.02	100.02	94.30	85.90	6.91
1	0.12	-	-	-	-	-	-	-	4.66
2	0.17	-	-	-	-	-	-	12.44	61.31
3	0.19	-	-	-	-	-	-	-	3.28
4	0.26	-	-	0.06	0.04	-	0.23	-	0.21
5	0.33	-	-	0.25	0.86	-	-	-	0.11
6	0.42	-	-	0.04	0.14	-	0.10	-	-
7	0.50	0.15	0.13	-	0.38	0.07	0.12	-	0.27
8	0.59	0.68	0.16	-	-	-	-	-	-
Imp. B	0.61	0.13	0.12	0.28	0.30	0.11	0.11	0.10	-
Imp. C/D	0.74	0.06	0.07	0.07	0.06	0.06	0.09	0.16	-
Imp. E	0.79	0.28	0.22	-	-	0.07	1.01	0.10	0.34
Imp. F	1.06	-	-	-	-	0.09	0.12	0.26	-
9	1.18	0.84	0.06	-	0.06	-	-	-	-
10	1.33	-	-	-	1.73	-	-	-	-
Imp. G	1.36	0.05	0.05	0.06	0.04	0.04	0.03	0.04	-
MB (%)		100.69	95.96	103.93	95.59	100.46	96.11	99.01	77.08

**Table 9:** Method feasibility study for the quantification of ITZ.

Parameter	Acceptance Criteria	Result	Conformity
System suitability (*n* = 6)	Area RSD ≤ 1.0% k’ ≥ 1.0 N ≥ 2000 Symmetry 0.8–1.8	0.92% 6.5 20,233 1.0	Complies
Stability	The area variation from the initial injection (initial time) must be ≤2.0%	Prepared solutions are stable for 48 h	-
Linearity (*n* = 5)	70 to 130 µg/mL R^2^ ≥ 0.999 b ≠ 0 Response Factor RSD ≤ 2.0%	70 to 130 µg/mL 0.9991 y = 19.066x + 83.749 1.3%	Complies
Accuracy (*n* = 9)	Mean recovery at 70, 100 and 130 µg/mL: 97.0–103.0%	70 µg/mL: 100.8% 100 µg/mL: 100.5% 130 µg/mL: 100.3%	Complies
Precision: Repeatability (*n* = 9)	Response Factor RSD ≤ 1.9% at 3 levels (70, 100 and 130 µg/mL) for reference solution and finished product	Reference: 0.5% Finished product: 1.9%	Complies
Intermediate precision (*n* = 6 per day)	Response Factor RSD ≤ 2.7% between two days preparation	Reference: 0.6% Finished product: 0.3%	Complies

**Table 10:** Method feasibility study for the quantification of related substances of ITZ.

Parameter	Acceptance Criteria	Result	Conformity
System suitability (*n* = 6)	Area RSD ≤ 5.0% k’ ≥ 1.0 N ≥ 2000 Symmetry 0.8–1.8	1.6% 6.6 20,644 1.0	Complies
Stability	The area variation from the initial injection (initial time) must be ≤2.0%	Prepared solutions are stable for 48 h	-
Linearity (*n* = 7)	LoQ to 1.3 µg/mL R^2^ ≥ 0.990 b ≠ 0 Response Factor RSD ≤ 5.0%	0.15 to 1.30 µg/mL 0.9909 y = 19.887x + 0.0933 3.7%	Complies
Accuracy (*n* = 9)	Mean recovery at LoQ, 1.0 and 1.3 µg/mL: 95.0–105.0%	LoQ: 102.6% 1.0 µg/mL: 100.5% 1.3 µg/mL: 99.7%	Complies
Precision: Repeatability (*n* = 9)	Response Factor RSD ≤ 5.0% at 3 levels (LoQ, 1.0 and 1.3 µg/mL) for reference solution and finished product	Reference: 3.6% Finished product: 3.6%	Complies
Intermediate precision (*n* = 6 per day)	Response Factor RSD ≤ 2.7% between two days preparation	Reference: 1.8%	Complies
LoD	S/N ≥ 3.0	3.7	Complies
LoQ	S/N ≥ 10.0 Area for 6 injections ≤ 5.0%	11.7 3.7%	Complies

**Table 11:** Comparison between different developed stability-indicating chromatographic methods for ITZ and the developed method in the present article [26,27,28,29].

Parameter	Developed Method	Kasagić et al., 2013 [26]	Agrawal et al., 2019 [27]	Reddy et al., 2025 [28]	Arghode et al., 2026 [29]
Flow (mL/min)	0.6	1.0	0.25	1.0	1.0
Mobile phase	ACN:BS (55:45)	ACN:H_2_O	ACN:BS	MeOH:HCl (99:1)	ACN:TEA (90:10)
Elution	Isocratic	Isocratic	Gradient	Isocratic	Isocratic
Run time (min.)	15	15	25	7	10
Diluent	ACN:BS (50:50)	MeOH:THF (50:50)	MeOH:ACN:BS (7:3:1)	MeOH:HCl (99:1)	MeOH (100)
Working Concentration (µg/mL)	100	200	400	200	30
Studied impurities	5	B and F	14 impurities	ND	5
LoD (µg/mL)	0.04	0.01–0.5	0.08	1.86	2.12
LoQ (µg/mL)	0.15	0.05–0.1	0.12	6.19	6.43
QbD	Yes	No	Yes	No	Yes

ND: Not determined.

**Table 12:** Comparison between the compendial chromatographic methods for ITZ and the developed method in the present article [4,5,6].

Parameter	Developed Method	EP (RS)	USP (Assay)	USP (RS)	USP Capsules (Assay)	USP Capsules (RS)
Flow (mL/min)	0.6	1.5	1.5	1.5	1.5	1.5
Mobile phase	ACN:BS (55:45)	ACN:BS	ACN:BS	ACN:BS	ACN+THF:BS (55:45)	ACN+THF:BS
Elution	Isocratic	Gradient	Gradient	Gradient	Isocratic	Gradient
Buffer solution	10 mM ammonium dihydrogen phosphate pH 4.0	80 mM tetrabutylammonium hydrogen sulphate	40 mM tetrabutylammonium hydrogen sulphate	80 mM tetrabutylammonium hydrogen sulphate	50 mM ammonium dihydrogen phosphate pH 2.0	50 mM ammonium dihydrogen phosphate pH 2.0
Run time (min.)	15	27	20	27	ND	50
Diluent	ACN:BS (50:50)	MeOH:HCl (99.6:0.4)	MeOH:HCl (99.6:0.4)	MeOH:HCl (99.6:0.4)	MeOH:THF (50:50)	MeOH:THF (50:50)
Working concentration	100	10,000	300	10,000	100	5000
AGREE score	0.57	0.47	0.49	0.47	0.48	0.44

**Table 13:** Sample preparation for the forced degradation study.

Stress Condition	Sample Preparation
Temperature	API solution: Weigh 10 mg of API and leave exposed to temperature at 105 °C for 72 h. Quantitatively transfer the stressed API to a 100.0 mL volumetric flask and dissolve in 80 mL of diluent. Sonicate for 10 min, allow to cool down and dilute to 100.0 mL with more diluent. Homogenise the solution with magnetic stirring. FP solution: Weigh 50 mg of FP, previously pulverised, and leave exposed to temperature at 105 °C for 72 h. Quantitatively transfer 50 mg of the stressed FP to a 100.0 mL volumetric flask and dissolve in 80 mL of diluent. Sonicate for 10 min, allow to cool down and dilute to 100.0 mL with more diluent. Homogenise the solution with magnetic stirring.
Acidic hydrolysis	API solution: Weigh 10 mg of API, transfer to a 100.0 mL volumetric flask, dissolve in 50 mL of diluent and sonicate for 5 min. Add 1.0 mL of hydrochloric acid 5 M and leave the preparation for 72 h. Neutralise the solution with 1.0 mL of sodium hydroxide 5 M. Sonicate for 10 min, allow to cool down and take it to the final volume with more diluent. Homogenise the solution with magnetic stirring. FP solution: Weigh 50 mg of FP, previously pulverised, transfer to a 100.0 mL volumetric flask, dissolve in 50 mL of diluent and sonicate for 5 min. Add 1.0 mL of hydrochloric acid 5 M and leave the preparation for 72 h. Neutralise the solution with 1.0 mL of sodium hydroxide 5 M. Sonicate for 10 min, allow to cool down and take it to the final volume with more diluent. Homogenise the solution with magnetic stirring.
Alkaline hydrolysis	API solution: Weigh 10 mg of API, transfer to a 100.0 mL volumetric flask, dissolve in 50 mL of diluent and sonicate for 5 min. Add 1.0 mL of sodium hydroxide 5 M and leave the preparation for 72 h. Neutralise the solution with 1.0 mL of hydrochloric acid 5 M. Sonicate for 10 min, allow to cool down and take it to the final volume with more diluent. Homogenise the solution with magnetic stirring. FP solution: Weigh 50 mg of FP, previously pulverised, transfer to a 100.0 mL volumetric flask, dissolve in 50 mL of diluent and sonicate for 5 min. Add 1.0 mL of sodium hydroxide 5 M and leave the preparation for 72 h. Neutralise the solution with 1.0 mL of hydrochloric acid 5 M. Sonicate for 10 min, allow to cool down and take it to the final volume with more diluent. Homogenise the solution with magnetic stirring.
Oxidative media	API solution: Weigh 10 mg of API, transfer to a 100.0 mL volumetric flask, dissolve in 50 mL of diluent and sonicate for 5 min. Add 1.0 mL of hydrogen peroxide 33% and leave the preparation for 24 h. Take it to the final volume with more diluent. Homogenise the solution with magnetic stirring. FP solution: Weigh 50 mg of FP, previously pulverised, transfer to a 100.0 mL volumetric flask, dissolve in 50 mL of diluent and sonicate for 5 min. Add 1.0 mL of hydrogen peroxide 33% and leave the preparation for 24 h. Take it to the final volume with more diluent. Homogenise the solution with magnetic stirring.

## Data Availability

The original contributions presented in the study are included in the article/Supplementary Materials, further inquiries can be directed to the corresponding author.

## Supplementary Materials

The following supporting information can be downloaded at: https://www.mdpi.com/article/10.3390/ph19071066/s1, Figure S1: Chromatogram corresponding to the acidic hydrolysis of itraconazole active ingredient after 72 h in stress conditions. The rest of the peaks, not identified with a name, correspond to the mobile phase and diluent used in the sample preparation and in the chromatographic method; Figure S2: Chromatogram corresponding to the acidic hydrolysis of the finished product containing itraconazole after 72 h in stress conditions. The rest of the peaks, not identified with a name, correspond to the mobile phase and diluent used in the sample preparation and in the chromatographic method and to the rest of the excipients of the pharmaceutical formulation; Figure S3: Chromatogram corresponding to the placebo solution obtained with the optimized chromatographic method by HPLC; Table S1: Developed and studied methods, specifying the HPLC parameters (I); Table S2: Developed and studied methods, specifying the HPLC parameters (II); Table S3: Developed and studied methods, specifying the HPLC parameters (III); Table S4: Developed and studied methods, specifying the HPLC parameters (IV); Table S5: Developed and studied methods, specifying the HPLC parameters (V); Table S6: Developed and studied methods, specifying the HPLC parameters (VI); Table S7: Developed and studied methods, specifying the HPLC parameters (VII); Table S8: Variance analysis (ANOVA) of the studied parameters in the Design of Experiments (DoE); Table S9: Conducted experiments in the first Design of Experiments (DoE) for the optimisation of the chromatographic method for the quantification of itraconazole and its degradation products; Table S10: Obtained results for the experiments conducted in the first Design of Experiments (DoE) for the optimisation of the chromatographic method for quantification of itraconazole and its degradation products; Table S11: Conducted experiments in the second Design of Experiments (DoE) for the optimisation of the chromatographic method for the quantification of itraconazole and its degradation products; Table S12: Obtained results for the experiments conducted in the second Design of Experiments (DoE) for the optimisation of the chromatographic method for the quantification of itraconazole and its degradation products; Table S13: Conducted experiments in the curvature approximation for the second Design of Experiments (DoE) for the optimisation of the chromatographic method for the quantification of itraconazole and its degradation products; Table S14: Obtained results for the experiments conducted in the curvature approximation for the second Design of Experiments (DoE) for the optimisation of the chromatographic method for quantification of itraconazole and its degradation products.

## Abbreviations

The following abbreviations are used in this manuscript:

ACN	Acetonitrile
API	Active pharmaceutical ingredient
ATP	Analytical Target Profile
BS	Buffer solution
CMA	Critical Method Attribute
DoE	Design of Experiments
EP	European Pharmacopoeia
FP	Finished product
HCl	Hydrochloric acid
HPLC	High-performance liquid chromatography
ITZ	Itraconazole
k’	Capacity factor
LoQ	Limit of quantification
LoD	Limit of detection
MeOH	Methanol
N	Number of theoretical plates
PVDF	Polyvinylidene fluoride
QbD	Quality by Design
RS	Related substances
Rs	Resolution
RSD	Relative standard deviation
SD	Standard deviation
SDM	Servei de Desenvolupament del Medicament
TEA	Triethylamine
THF	Tetrahydrofuran
USP	Unites States Pharmacopoeia
