# Supplementary material for: Development and Optimisation of a Stability-Indicating Analytical Method for Itraconazole in Drug Product by High-Performance Liquid Chromatography Using a QbD Approach
Source: Pharmaceuticals (Basel). 2026 Jul 10;19(7):1066. doi: 10.3390/ph19071066 (PMC13414520; doi:10.3390/ph19071066)
Supplement: Supplementary file 1 [file pharmaceuticals-19-01066-s001.zip › pharmaceuticals-4309753-supplementary.pdf]

Development and Optimisation of a Stability-Indicating Analytical Method for Itraconazole in Drug Product by High-Performance Liquid Chromatography Using a QbD Approach

Alex Fraschi-Nieto [1], Xavier Mula-Roldán [1], Lluís Gavaldà-Cánovas [1], Débora Mercadé-Frutos [1], Encarna García-Montoya [1,2], Marc Suñé-Pou [1,2,*] and Pilar Pérez-Lozano [1,2]

[1] Department of Pharmacy and Pharmaceutical Technology and Physical Chemistry, Faculty of Pharmacy, University of Barcelona, Av. Joan XXIII, 27-31, 08028 Barcelona, Spain; alexfraschi@ub.edu (A.F.-N.); xavimula@ub.edu (X.M.-R.); ll.gavalda.c@ub.edu (L.G.-C.); debora.mercade@ub.edu (D.M.-F.); encarnagarcia@ub.edu (E.G.-M.); perezlo@ub.edu (P.P.-L.)

[2] Pharmacotherapy, Pharmacogenetics and Pharmaceutical Technology Research Group, Bellvitge Biomedical Research Institute (IDIBELL), Av. Gran Via de l'Hospitalet, 199-203, 08090 L'Hospitalet de Llobregat, Spain

* Correspondence: marcsune@ub.edu; Tel.: +34-93-402-45-48

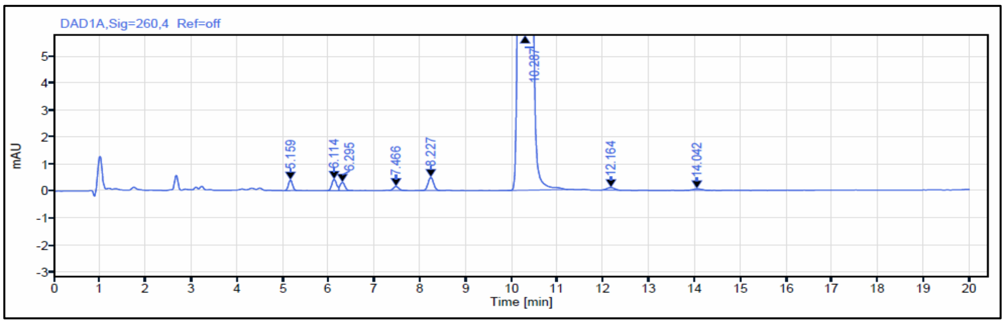

Figure S1. Chromatogram corresponding to the acidic hydrolysis of itraconazole active ingredient after 72 hours in stress conditions. The rest of the peaks, not identified with a name, corresponds to the mobile phase and diluent used in the sample preparation and in the chromatographic method.

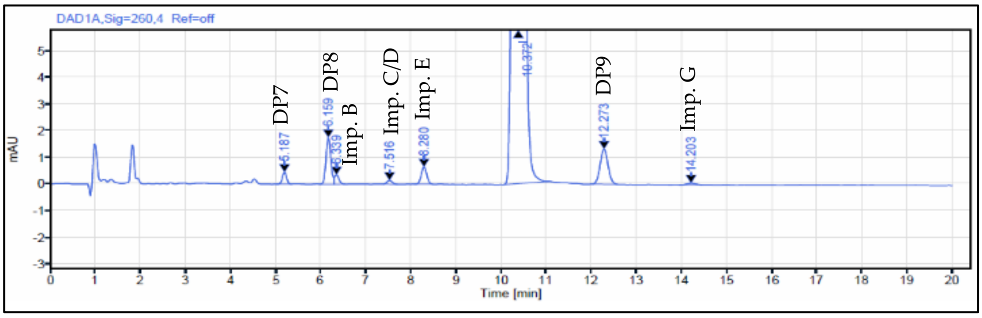

Figure S2. Chromatogram corresponding to the acidic hydrolysis of the finished product containing itraconazole after 72 hours in stress conditions. The rest of the peaks, not identified with a name, corresponds to the mobile phase and diluent used in the sample preparation and in the chromatographic method and to the rest of the excipients of the pharmaceutical formulation.

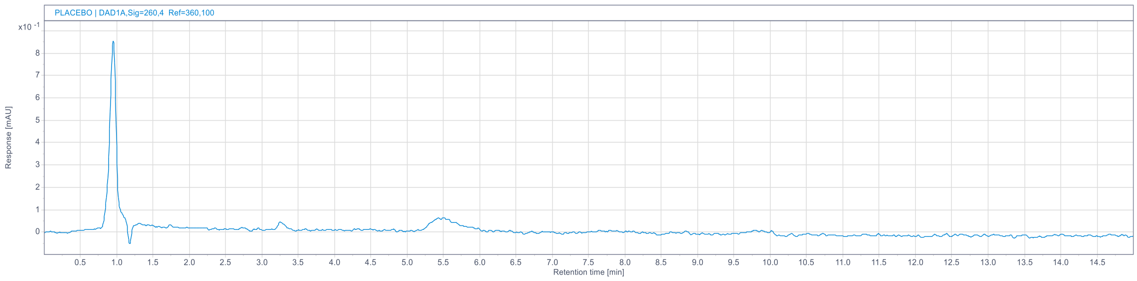

Figure S3. Chromatogram corresponding to the placebo solution obtained with the optimized chromatographic method by HPLC.

Table S1. Developed and studied methods, specifying the HPLC parameters (I)

Parameter	ITRACO01	ITRACO02	ITRACO03
Stationary phase	Zorbax C18 100x4.6mm, 3.5 µm	Zorbax C18 100x4.6mm, 3.5 µm	Zorbax C18 100x4.6mm, 3.5 µm
Mobile phase	ACN:BS (Gradient)	ACN:BS (Gradient)	ACN:BS (Gradient)
BS	67 mM Tetrabutylammonium hydrogen sulphate	10 mM Ammonium dihydrogen phosphate (pH 2.0)	10 mM Ammonium dihydrogen phosphate (pH 2.0)
Flow rate	1.5 mL/min	1.5 mL/min	1.5 mL/min
Wavelength	225 nm	260 nm	260 nm
Inj. volume	10 µL	10 µL	5 to 50 µL
Temperature	30 °C	30 °C	30 °C
Diluent	Methanolic HCl	Methanolic HCl	Methanolic HCl
Concentration	300 µg/mL	300 µg/ mL	100, 200, 300 and 500 µg/ mL

Table S2. Developed and studied methods, specifying the HPLC parameters (II)

Parameter	ITRACO04	ITRACO04A	ITRACO04B
Stationary phase	Zorbax C18 100x4.6mm, 3.5 µm	Zorbax C18 100x4.6mm, 3.5 µm	Zorbax C18 100x4.6mm, 3.5 µm
Mobile phase	ACN:BS (50:50)	ACN:BS (45:55)	ACN:BS (40:60)
BS	10 mM Ammonium dihydrogen phosphate (pH 2.0)	10 mM Ammonium dihydrogen phosphate (pH 2.0)	10 mM Ammonium dihydrogen phosphate (pH 2.0)
Flow rate	1.0 mL/min	1.0 mL/min	1.0 mL/min
Wavelength	260 nm	260 nm	260 nm
Inj. volume	10 µL	10 µL	10 µL
Temperature	30 °C	30 °C	30 °C
Diluent	Methanolic HCl	Methanolic HCl	Methanolic HCl
Concentration	100 µg/mL	100 µg/mL	100 µg/mL

Table S3. Developed and studied methods, specifying the HPLC parameters (III)

Parameter	ITRACO05	ITRACO06	ITRACO06A
Stationary phase	XSELECT HSS T3 C18 250x4.6 mm 3.5 µm	SEA C18, 150x4.6 mm, 3.0 µm	SEA C18, 150x4.6 mm, 3.0 µm
Mobile phase	ACN:BS (50:50)	ACN:BS (50:50)	ACN:BS (55:45)
BS	10 mM Ammonium dihydrogen phosphate (pH 2.5)	10 mM Ammonium dihydrogen phosphate (pH 2.5)	10 mM Ammonium dihydrogen phosphate (pH 2.5)
Flow rate	1.0 mL/min	1.0 mL/min	1.0 mL/min
Wavelength	260 nm	260 nm	260 nm
Inj. volume	10, 15, 20, 25, 30, 40 and 50 µL	20 µL	20 µL
Temperature	30 °C	30 °C	30 °C
Diluent	Mobile phase	Mobile phase	Mobile phase
Concentration	100 and 1 µg/mL	100 µg/mL	100 µg/mL

Table S4. Developed and studied methods, specifying the HPLC parameters (IV)

Parameter	ITRACO06B	ITRACO06C	ITRACO06D
Stationary phase	SEA C18, 150x4.6 mm, 3.0 µm	SEA C18, 150x4.6 mm, 3.0 µm	SEA C18, 150x4.6 mm, 3.0 µm
Mobile phase	ACN:BS (50:50)	ACN:BS (52:48)	ACN:BS (50:50)
BS	10 mM Ammonium dihydrogen phosphate (pH 2.5)	10 mM Ammonium dihydrogen phosphate (pH 2.5)	10 mM Ammonium dihydrogen phosphate (pH 2.5)
Flow rate	1.5 mL/min	1.0 mL/min	1.2 mL/min
Wavelength	260 nm	260 nm	260 nm
Inj. volume	20 µL	20 µL	20 µL
Temperature	30 °C	30 °C	30 °C
Diluent	Mobile phase	Mobile phase	Mobile phase
Concentration	100 µg/mL	100 µg/mL	100 µg/mL

Table S5. Developed and studied methods, specifying the HPLC parameters (V)

Parameter	ITRACO07	ITRACO08	ITRACO09
Stationary phase	ODS Hypersil 100x4.6 mm, 3.0 µm	ODS Hypersil 100x4.6 mm, 3.0 µm	Luna Phenyl-Hexyl 150x4.6 mm, 5 µm
Mobile phase	ACN:BS (50:50)	ACN:BS (50:50)	ACN:BS (50:50)
BS	100 mM Ammonium dihydrogen phosphate (pH 2.5)	100 mM Ammonium dihydrogen phosphate (pH 2.5)	10 mM Ammonium dihydrogen phosphate (pH 2.5)
Flow rate	1.5 mL/min	1.2 mL/min	1.5 mL/min
Wavelength	260 nm	260 nm	260 nm
Inj. volume	20 µL	20 µL	20 µL
Temperature	30 °C	30 °C	30 °C
Diluent	Mobile phase	Mobile phase	Mobile phase
Concentration	100 µg/mL	100 µg/mL	100 µg/mL

Table S6. Developed and studied methods, specifying the HPLC parameters (VI)

Parameter	ITRACO10	ITRACO11	ITRACO12
Stationary phase	Dionex Acclaim C18 33x3.0 mm, 3.0 µm	Acquity CSH C18 100x3.0 mm, 1.7 µm	Poroshell EC-C18 100x4.6 mm, 2.7 µm
Mobile phase	ACN:BS (50:50)	ACN:BS (50:50)	ACN:BS (50:50)
BS	10 mM Ammonium dihydrogen phosphate (pH 2.5)	10 mM Ammonium dihydrogen phosphate (pH 2.5)	10 mM Ammonium dihydrogen phosphate (pH 2.5)
Flow rate	1.5 mL/min	0.25 mL/min	1.0 mL/min
Wavelength	260 nm	260 nm	260 nm
Inj. volume	20 µL	10 µL	15 µL
Temperature	30 °C	50 °C	50 °C
Diluent	Mobile phase	Mobile phase	Mobile phase
Concentration	100 µg/mL	100 µg/mL	100 µg/mL

Table S7. Developed and studied methods, specifying the HPLC parameters (VII)

Parameter	ITRACO13	ITRACO14	ITRACO15
Stationary phase	Poroshell EC-C18 100x4.6 mm, 2.7 µm	Poroshell EC-C18 100x4.6 mm, 2.7 µm	Poroshell EC-C18 150x3.0 mm, 2.7 µm
Mobile phase	ACN:BS (50:50)	ACN:BS (50:50)	ACN:BS (55:45)
BS	10 mM Ammonium dihydrogen phosphate (pH 2.5)	10 mM Ammonium dihydrogen phosphate (pH 2.5)	10 mM Ammonium dihydrogen phosphate (pH 2.5)
Flow rate	1.2 mL/min	1.5 mL/min	0.5 mL/min
Wavelength	260 nm	260 nm	260 nm
Inj. volume	15 µL	15 µL	5 µL
Temperature	50 °C	50 °C	50 °C
Diluent	Mobile phase	Mobile phase	ACN:BS (50:50)
Concentration	100 µg/mL	100 µg/mL	100 µg/mL

Table S8. Variance analysis (ANOVA) of the studied parameters in the Design of Experiments (DoE).

Parameter	Retention time		Resolution	
	F-value	p-value	F-value	p-value
**Lineal**				
Flow rate	30,718.46	0.000	244.75	0.000
Mobile phase	31,294.29	0.000	2,009.16	0.000
BS pH	76.06	0.000	183.14	0.000
BS concentration	31.66	0.000	99.58	0.000
Column	1228.61	0.000	776.07	0.000
**2 terms interaction**				
Flow rate * Mobil phase	5445.33	0.000	1,029.97	0.000
Flow rate * BS pH	86.62	0.000	8.44	0.008
Flow rate * BS concentration	44.51	0.000	70.56	0.000
Flow rate * Column	2,476.09	0.000	61.24	0.000
Mobile phase * BS pH	91.39	0.000	5.46	0.029
Mobile phase * BS concentration	3.46	0.077	100.40	0.000
Mobile phase * Column	4,975.05	0.000	1,731,08	0.000
BS pH * BS concentration	817.83	0.000	2,764.34	0.000
BS pH * Column	7.11	0.014	4.65	0.043
BS concentration * Column	86.27	0.000	70.70	0.000
**4 terms interaction**				
Flow rate * Mobile phase * BS pH * BS concentration	968.36	0.000	7.23	0.014
**Lack-of-fit**	17.36	0.007	1.67	0.332

Table S9. Conducted experiments in the first Design of Experiments (DoE) for the optimization of the chromatographic method for the quantification of itraconazole and its degradation products.

Experiment (number)	Flow (mL/min)	Mobile phase proportion (ACN:BS)	Buffer solution pH	Buffer solution concentration (mM)	Column
1	0.3	60:40	3.0	15	441[1]
2	0.7	50:50	5.0	5	441
3	0.5	55:45	4.0	10	441
4	0.3	60:40	3.0	5	428[2]
5	0.3	60:40	5.0	15	428
6	0.3	50:50	5.0	15	441
7	0.7	60:40	3.0	15	428
8	0.7	50:50	3.0	5	428
9	0.3	50:50	3.0	5	441
10	0.5	55:45	4.0	10	428
11	0.7	50:50	3.0	15	441
12	0.5	55:45	4.0	10	428
13	0.7	60:40	5.0	15	441
14	0.7	60:40	5.0	5	428
15	0.3	50:50	5.0	5	428
16	0.7	50:50	5.0	15	428
17	0.7	60:40	3.0	5	441
18	0.5	55:45	4.0	10	441
19	0.3	60:40	5.0	5	441
20	0.3	50:50	3.0	15	428
21	0.7	60:40	5.0	15	441
22	0.3	60:40	5.0	5	441
23	0.3	50:50	5.0	5	428
24	0.5	55:45	4.0	10	428
25	0.7	50:50	5.0	5	441
26	0.7	50:50	5.0	15	428
27	0.7	50:50	3.0	5	428
28	0.3	60:40	3.0	5	428
29	0.3	60:40	5.0	15	428
30	0.5	55:45	4.0	10	441
31	0.7	60:40	3.0	15	428
32	0.5	55:45	4.0	10	428
33	0.7	50:50	3.0	15	441
34	0.3	50:50	3.0	5	441
35	0.5	55:45	4.0	10	441
36	0.3	50:50	3.0	15	428
37	0.3	50:50	5.0	15	441
38	0.7	60:40	3.0	5	441
39	0.7	60:40	5.0	5	428
40	0.3	60:40	3.0	15	441

[1]441 is the codification referring to the chromatographic column Poroshell EC-C18 100 x 4.6 mm 2.7 μm

[2]428 is the codification referring to the chromatographic column Poroshell EC-C18 150 x 3.0 mm 2.7 μm

Table S10. Obtained results for the experiments conducted in the first Design of Experiments (DoE) for the optimization of the chromatographic method for quantification of itraconazole and its degradation products.

Experiment (number)	Resolution between ITZ and Impurity F	Peak symmetry	Retention time (minutes)
1	1.15459	*	6.413
2	1.11579	0.9578	5.582
3	1.84119	0.9388	6.595
4	2.40841	0.9296	11.442
5	2.21928	1.0558	11.265
6	2.56325	0.9675	13.156
7	1.61793	0.9194	4.865
8	3.39901	0.9642	12.964
9	2.71275	0.9937	16.104
10	2.73366	1.0536	10.416
11	1.16173	*	5.646
12	2.73501	1.0537	10.316
13	2.00215	*	2.376
14	1.67992	1.0117	4.909
15	3.19668	1.0521	29.612
16	3.33396	1.0388	12.796
17	2.93347	0.8837	2.306
18	1.85183	0.9246	6.547
19	1.31963	*	6.727
20	3.61772	1.0375	30.736
21	2.01861	1.0498	2.345
22	1.22614	1.0375	6.965
23	3.25239	1.0470	29.524
24	2.71389	1.0498	10.289
25	1.21004	0.9246	5.527
26	3.19797	1.0466	12.583
27	3.51819	0.9865	12.184
28	2.50362	0.9578	11.801
29	2.23933	1.0504	11.236
30	1.86889	0.9391	6.590
31	1.58911	0.9280	4.886
32	2.77033	1.0470	10.267
33	1.14763	1.0497	5.627
34	2.76923	0.9930	16.170
35	1.78175	0.9349	6.593
36	3.57579	1.0163	30.540
37	2.58135	0.9687	13.387
38	2.95741	0.8862	2.298
39	1.63638	1.0048	4.908
40	1.23293	*	6.362

*Not determinated.

Table S11. Conducted experiments in the second Design of Experiments (DoE) for the optimization of the chromatographic method for the quantification of itraconazole and its degradation products.

Experiment (number)	Flow (mL/min)	Mobile phase proportion (ACN:BS)	Buffer solution pH
1	0.4	50:50	4.5
2	0.6	50:50	4.5
3	0.6	60:400	4.5
4	0.4	60:400	3.5
5	0.5	55:45	4.0
6	0.4	60:400	4.5
7	0.5	55:45	4.0
8	0.5	55:45	4.0
9	0.6	60:400	3.5
10	0.6	50:50	3.5
11	0.4	50:50	3.5
12	0.6	50:50	4.5
13	0.6	50:50	3.5
14	0.4	60:400	3.5
15	0.6	60:400	3.5
16	0.4	60:400	4.5
17	0.5	55:45	4.0
18	0.4	50:50	4.5
19	0.5	55:45	4.0
20	0.6	60:400	4.5
21	0.4	50:50	3.5
22	0.5	55:45	4.0

Table S12. Obtained results for the experiments conducted in the second Design of Experiments (DoE) for the optimization of the chromatographic method for the quantification of itraconazole and its degradation products.

Experiment (number)	Resolution	Retention time (minutes)
1	2.78948	21.172
2	2.82546	14.867
3	1.50321	5.494
4	1.55003	8.129
5	2.34072	10.164
6	1.51215	8.145
7	2.29409	10.141
8	2.31515	10.200
9	1.54341	5.504
10	2.84418	14.681
11	2.64015	21.268
12	2.70868	14.879
13	2.82787	14.607
14	1.55211	8.117
15	1.50710	5.507
16	1.53953	8.122
17	2.28918	10.187
18	2.81746	21.676
19	2.35115	10.254
20	1.51124	5.483
21	2.81466	21.241
22	2.42635	10.861

Table S13. Conducted experiments in the curvature approximation for the second Design of Experiments (DoE) for the optimization of the chromatographic method for the quantification of itraconazole and its degradation products.

Experiment (number)	Flow (mL/min)	Mobile phase proportion (ACN:BS)	Buffer solution pH
1	0.4	50:50	4.5
2	0.6	50:50	4.5
3	0.6	60:40	4.5
4	0.4	60:40	3.5
5	0.5	55:45	4.0
6	0.4	60:40	4.5
7	0.5	55:45	4.0
8	0.5	55:45	4.0
9	0.6	60:40	3.5
10	0.6	50:50	3.5
11	0.4	50:50	3.5
12	0.6	50:50	4.5
13	0.6	50:50	3.5
14	0.4	60:40	3.5
15	0.6	60:40	3.5
16	0.4	60:40	4.5
17	0.5	55:45	4.0
18	0.4	50:50	4.5
19	0.5	55:45	4.0
20	0.6	60:40	4.5
21	0.4	50:50	3.5
22	0.5	55:45	4.0
23	0.4	55:45	4.0
24	0.5	55:45	4.0
25	0.5	50:50	4.0
26	0.5	55:45	3.5
27	0.5	55:45	4.0
28	0.5	55:45	4.5
29	0.5	60:40	4.0
30	0.6	55:45	4.0
31	0.5	55:45	4.0

Table S14. Obtained results for the experiments conducted in the curvature approximation for the second Design of Experiments (DoE) for the optimization of the chromatographic method for quantification of itraconazole and its degradation products.

Experiment (number)	Resolution	Retention time (minutes)
1	2.78948	21.172
2	2.82546	14.867
3	1.50321	5.494
4	1.55003	8.129
5	2.34072	10.164
6	1.51215	8.145
7	2.29409	10.141
8	2.31515	10.200
9	1.54341	5.504
10	2.84418	14.681
11	2.64015	21.268
12	2.70868	14.879
13	2.82787	14.607
14	1.55211	8.117
15	1.50710	5.507
16	1.53953	8.122
17	2.28918	10.187
18	2.81746	21.676
19	2.35115	10.254
20	1.51124	5.483
21	2.81466	21.241
22	2.42635	10.861
23	2.46745	13.507
24	2.33852	10.276
25	2.72671	17.397
26	2.34821	10.231
27	2.34470	10.278
28	2.39430	10.250
29	1.52072	6.636
30	2.23484	8.711
31	2.29717	10.259
